# 18F-FDG PET/CT: an unexpected case of Huntington’s disease

**DOI:** 10.1186/s12883-019-1311-9

**Published:** 2019-05-01

**Authors:** Sebastian Michels, Hans-Georg Buchholz, Florian Rosar, Isabel Heinrich, Manuela A. Hoffmann, Susann Schweiger, Oliver Tüscher, Mathias Schreckenberger

**Affiliations:** 1 Department of Nuclear Medicine, University Medical Center of Johannes Gutenberg-University, Mainz, Germany; 2 Department of Psychiatry and Psychotherapy, University Medical Center of the Johannes Gutenberg-University, Mainz, Germany; 3 Department of Human Genetics, University Medical Center of Johannes Gutenberg-University, Mainz, Germany; 4 Centre for Rare Diseases of the Central Nervous System, University Medical Center of the Johannes Gutenberg-University, Mainz, Germany

**Keywords:** Huntington’s disease, FDG-PET/CT, Striatal hypometabolism

## Abstract

**Background:**

Huntington’s disease (HD) is a rare, genetic neurodegenerative disorder often presenting with emotional, cognitive and behavioral abnormalities before manifestation of disease defining motor symptoms. Cognitive impairment is a frequent clinical feature caused by different dementia subtypes. Imaging cortical and subcortical glucose metabolism via ^18^F-FDG PET/CT can help to discriminate the underlying disease.

**Case presentation:**

The patient is a 54-year old man presenting with progressive cognitive impairment and mild orofacial dyskinesia. ^18^F-FDG PET/CT of the brain revealed a severe bilateral hypometabolism in the striatum. Following imaging Huntington’s disease was suspected and a molecular genetic testing confirmed the diagnosis.

**Conclusions:**

Huntington’s disease is a rare but important differential diagnosis of cognitive impairment, especially before motor symptoms are manifest. ^18^F-FDG PET is capable to show early striatal dysfunction in HD even when structural imaging is normal. We conclude that, in cases with negative family history the HD characteristic metabolic pattern can lead to the diagnosis when no other dementia-suspected changes are present.

## Background

^18^F-FDG PET/CT studies of the brain are nowadays a common imaging technique to reveal the underlying cause of cognitive impairment at an early stage. The prevalence of dementia subtypes in different surveys varies depending on sociodemographic aspects and diagnostic workup (i.e. confirmed histopathologically or clinically) [1, 2]. Alzheimer’s dementia is the most frequent subtype, followed by vascular dementia, frontotemporal dementia and Lewy body dementia. Other dementia subtypes include a vast heterogeneity of syndromes, such as Creutzfeld-Jakob disease, alcohol abuse and different Parkinson syndromes amongst others.

Imaging glucose metabolism via ^18^F-FDG PET is suitable for identifying different patterns of cortical hypometabolism, which have proven a high diagnostic accuracy in predicting conversion from mild cognitive impairment to a specific type of dementia [3].

## Case presentation

A 54-year old man presenting with progressive cognitive impairment was admitted to the memory clinic at the Department of Psychiatry and Psychotherapy. His Mini-Mental State Examination score showed a cognitive impairment (24 out of 30 points). Furthermore, he had mild orofacial dyskinesia, which was suggested to be a side-effect of his medication with melperone. The patient received this treatment because of a sleep disorder and restlessness. After clinical examination he was referred to the Department of Nuclear Medicine with the suspected diagnosis of Alzheimer’s disease for an ^18^F-FDG PET/CT study. Family history was empty showing no neurodegenerative or psychiatric diseases in first degree relatives. The patient’s father had suffered from a stroke with persisting disability and died at the age of 66 years.

**Fig. 1:**
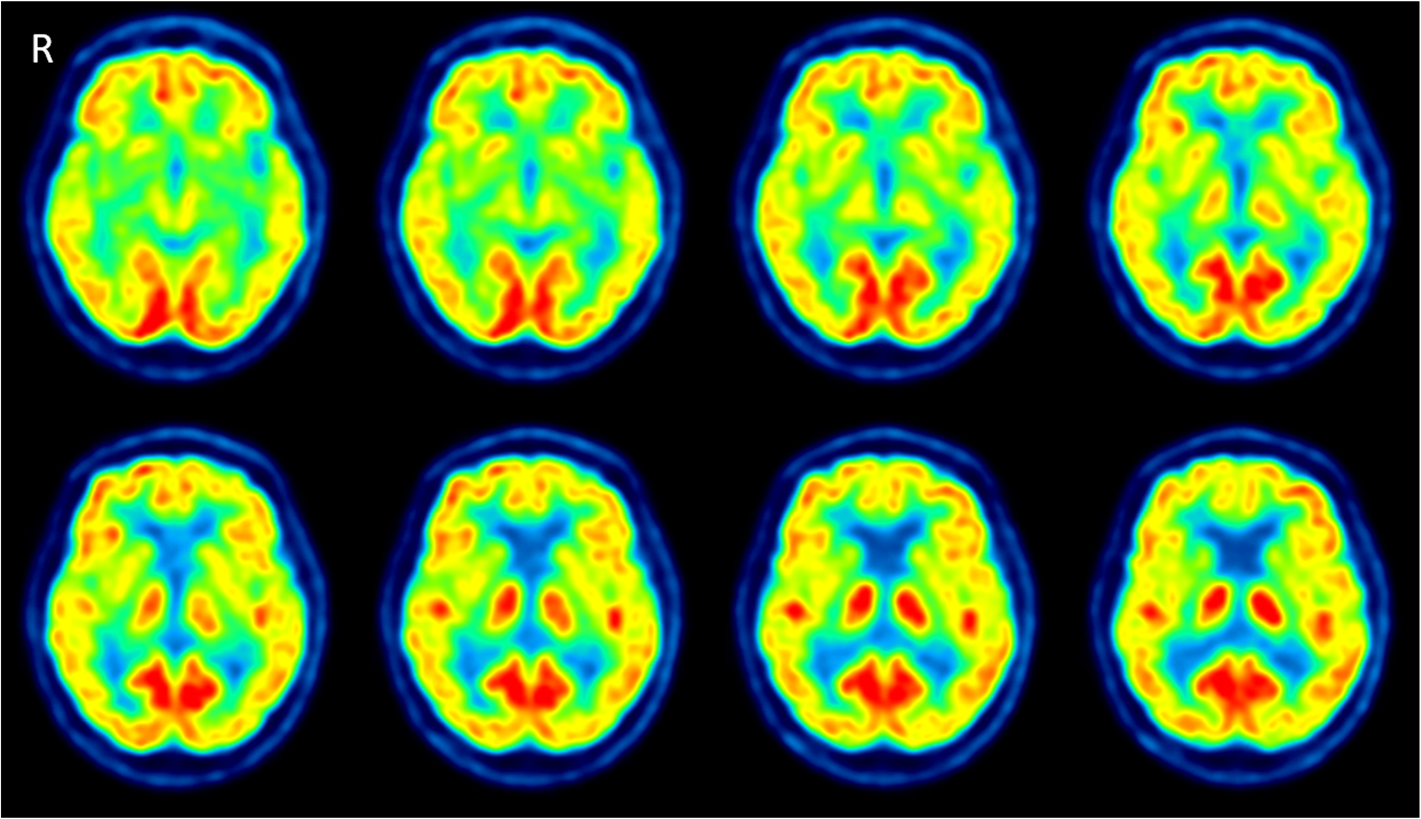
Axial images of ^18^F-FDG PET/CT scan demonstrating the severed striatal hypometabolism

**Fig. 2:**
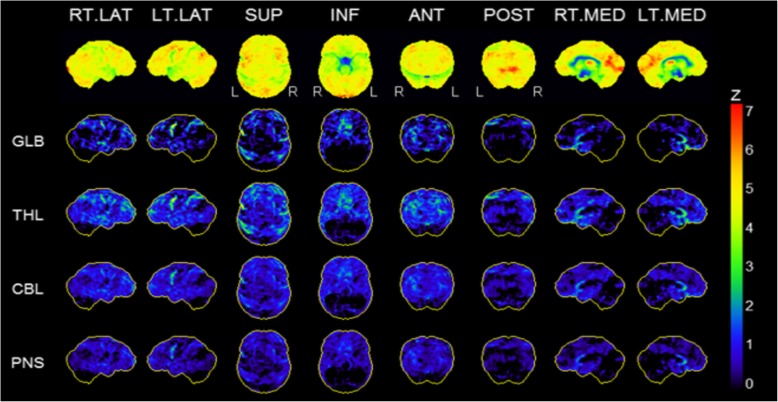
Statistical stereotactic surface projection maps showing no significant cortical hypometabolism

**Fig. 3:**
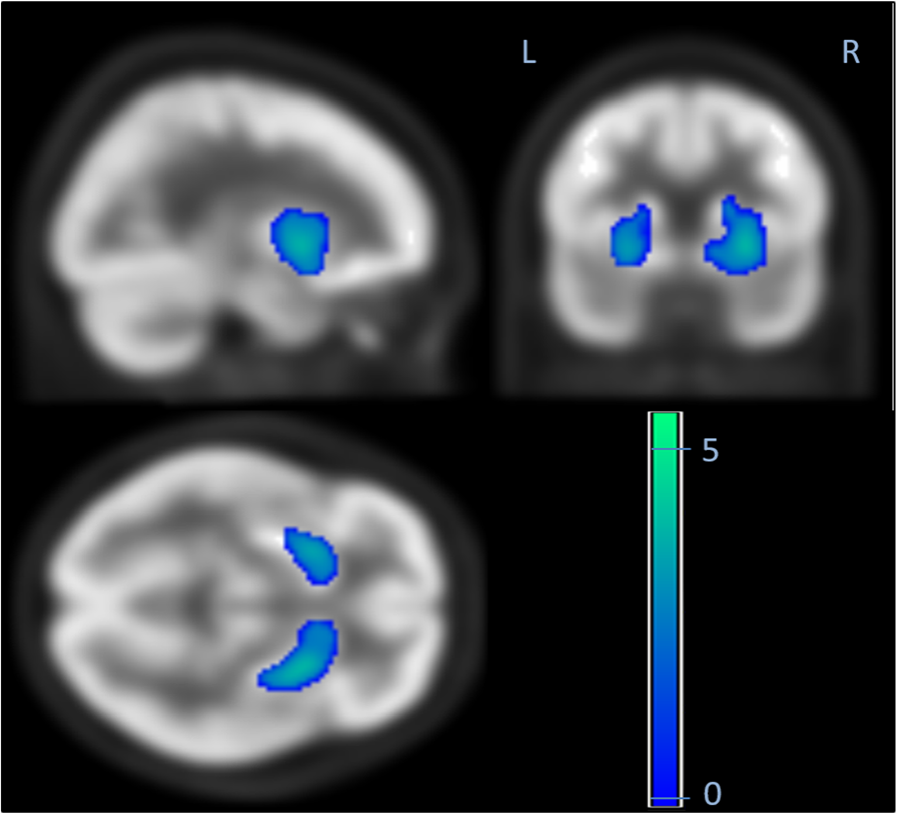
Voxel-by-voxel single subject analysis showing striatal hypometabolism

While CT and MRI showed no pathologic findings, the ^18^F-FDG PET/CT (Philips Gemini TF16, Best, Netherlands) of the brain revealed a severe bilaterally decreased uptake in the striatum (Fig. 1). Additionally, we performed two different voxel-based intersubject statistical analyses to a reference database. First, using NEUROSTAT [4] we performed 3D standard surface projections (3D-SSP) from patient’s ^18^F-FDG-PET and compared them to an age-matched 3D-SSP database in order to detect dementia-related hypometabolism in the cortical areas (Fig. 2). In a second step, focusing on subcortical regions, we used SPM 12 (Wellcome Trust Centre for Neuroimaging at UCL, London) implemented in Matlab 9.0 (MathWork, Sherborn, Mass.) and performed a voxel-by-voxel single subject analysis of the whole brain. We then compared our patient to an age-matched healthy group used in NEUROSTAT [5]. Concordant to the visual findings we found a significant reduced bilateral uptake in the striatum (Fig. 3).

Following imaging Huntington’s disease (HD) was being favored as underlying cause for the patient’s cognitive impairment. Subsequently, a molecular genetic testing was initiated, which confirmed the diagnosis of HD. One allele showed an abnormal CAG repeat of the Huntingtin gene (HTT) in chromosome 4p16.3 of 41 ± 1, while the other allele was normal (CAG repeat of 20 ± 1).

## Discussion and conclusion

Even though patterns of cortical metabolism are an accurate surrogate parameter for the majority of different dementia subtypes, there are some less common disorders associated with cognitive impairment showing primarily subcortical abnormalities and no cortical alterations.

When evaluating cognitive impairment with striatal hypometabolism different diagnoses come to mind. For example, various tauopathies like progressive supranuclear palsy and corticobasal degeneration. They share striatal involvement resulting in hypometabolism and progressive dementia as a core feature. However they usually present with additional cortical hypometabolism even in early stages; in addition the patient showed no evidence of an akinetic movement disorder.

On the other hand, different genetic disorders affecting the basal ganglia in combination with cognitive decline can be considered. Based on their prevalence Wilson’s and Huntington’s disease are probable differential diagnosis. Furthermore, some even more rare entities including Dentatorubral-pallidoluysian atrophy, a subtype of spinocerebellar degeneration, can also be associated with cognitive impairment and striatal abnormalities/hypometabolism [6].

Wilson’s disease (WD), an autosomal-recessive condition resulting in accumulation of copper in brain and liver can present with initially neuropsychiatric symptoms including dementia often in combination with parkinsonism or dystonia. FDG-PET scans in WD have shown – consistent with our case - a decreased striatal uptake (mainly in the caudate nucleus and in the lenticular nuclei) while the cortical glucose metabolism often remains normal [7, 8]. Nevertheless, onset of WD > 40 years is rare and no Kayser-Fleischer ring as a pathognomonic sign of copper deposition in the cornea could be identified in the patient. Moreover, liver function tests were normal.

Based on the PET scan HD was being suggested as underlying cause for the cognitive impairment despite the fact that the patient had no family history of HD. Only ^18^F-FDG PET revealed an abnormal striatal function. Interestingly, we found no alterations of cortical metabolism or changes in structural neuroimaging. Although early-stage HD has been associated with frontotemporal and parietal hypometabolism [9] and progressive atrophy of the striatum on MRI [10].

HD is an autosomal-dominant neurodegenerative disorder caused by an increase in a trinucleotide repeat section in the HTT. When the repeat count of the CAG sequence reaches a certain threshold (CAG repeats > 35) a mutant form of the Huntingtin protein is produced which is neurotoxic. CAG repeats > 39 always lead to the onset of disease, whereas CAG repeats between 36 and 39 are associated with a reduced penetrance of the disorder [11]. The age at clinical onset is negatively correlated with the CAG length. Typical age of onset of disease is the 4th -5th decade, whereas up to 10% of patients present with an onset > 60 years of age [12]. Genetic testing is generally initiated due to typical movement aberrations in patients and a positive family history in first degree relatives. However, cognitive impairment often precedes the clinical onset of motor symptoms [13]. Interestingly, cognitive symptoms in HD are more dominant in men than in women with stronger contribution to patients’ independence and functioning [14].

Severely reduced glucose metabolism in the bilateral striatum is a well-described (and characteristic) neuroimaging finding in HD [15]. Due to genetic testing as the current routine standard in the HD diagnostic pathway, FDG-PET is usually not performed in patients with suspected HD. However, HD is a rare but important differential diagnosis of cognitive impairment, especially before motor symptoms are manifest. We conclude that in cases with negative family history the HD characteristic metabolic pattern can lead to the diagnosis when no other dementia-suspected (cortical) changes are present.

## Abbreviations

^18^F-FDG PET/CT: 18F-fludeoxyglucose positron emission tomography/computer tomography
3D-SSP: 3D standard surface projections
CAG: Cytosine-adenine-guanine
CT: Computer tomography
HD: Huntington’s disease
HTT: Huntingtin gene
MRI: Magnetic resonance imaging
WD: Wilson’s disease
